# Metformin Alleviates Diabetes-Associated Hypertension by Attenuating the Renal Epithelial Sodium Channel

**DOI:** 10.3390/biomedicines11020305

**Published:** 2023-01-21

**Authors:** Yogesh M. Scindia, Mohammed F. Gholam, Alina Waleed, Lauren P. Liu, Kevin M. Chacko, Dhruv Desai, Juliana Pena Lopez, Zeeshan Malik, Whitney C. Schramm, Angelica G. Morales, Morgan Carson-Marino, Abdel A. Alli

**Affiliations:** 1 Division of Pulmonary, Critical Care and Sleep Medicine, Department of Medicine, College of Medicine, University of Florida, Gainesville, FL 32610, USA; 2 Division of Nephrology, Hypertension and Renal Transplantation, Department of Medicine, College of Medicine, University of Florida, Gainesville, FL 32610, USA; 3 Department of Pathology, University of Florida, Gainesville, FL 32610, USA; 4 Department of Physiology and Aging, College of Medicine, University of Florida, Gainesville, FL 32610, USA; 5 Department of Basic Medical Sciences, College of Medicine, King Saud bin Abdulaziz University for Health Sciences, Jeddah 21423, Saudi Arabia; 6 Department of Pharmaceutics, College of Pharmacy, University of Florida, Gainesville, FL 32610, USA

**Keywords:** diabetes, hypertension, ENaC, cathepsin B

## Abstract

Diabetic nephropathy is the primary cause of morbidity in type 2 diabetes mellitus (T2DM) patients. New data indicate that hypertension, a common comorbidity in T2DM, can worsen outcomes of diabetic nephropathy. While metformin is a commonly prescribed drug for treating type 2 diabetes, its blood pressure regulating ability is not well documented. The aim of this study was to investigate the effect of metformin on normalizing blood pressure in salt-loaded hypertensive diabetic db/db mice. Sixteen-week-old male and female diabetic db/db mice were individually placed in metabolic cages and then randomized to a control vehicle (saline) or metformin treatment group. We evaluated the blood pressure reducing ability of metformin in salt-induced hypertension and progression of nephropathy in db/db mice. We observed that metformin- normalized systolic blood pressure in hypertensive diabetic mice. Mechanistically, metformin treatment reduced renal cathepsin B expression. Low cathepsin B expression was associated with reduced expression and activity of the epithelial sodium channel (ENaC), sodium retention, and thus control of hypertension. In addition, we identified that urinary extracellular vesicles (EVs) from the diabetic mice are enriched in cathepsin B. Compared to treatment with urinary EVs of vehicle-treated hypertensive diabetic mice, the amiloride-sensitive transepithelial current was significantly attenuated upon exposure of renal collecting duct cells to urinary EVs isolated from metformin-treated db/db mice or cathepsin B knockout mice. Collectively, our study identifies a novel blood pressure reducing role of metformin in diabetic nephropathy by regulating the cathepsin B-ENaC axis.

## 1. Introduction

Type 2 diabetes mellitus (T2DM) is a metabolic disease, and represents 7.7% of the total population aged 20–79 years [1]. Although improvements have been made in treating diabetes and its kidney and cardiovascular complications, the risk of morbidity and mortality remains high [2,3]. T2DM and hypertension commonly coexist [4,5,6]. The coexistence of T2DM and hypertension confers an increased risk of cardiovascular disease, chronic kidney disease (CKD), and death compared with normotensive and nondiabetic adults [5]. However, factors contributing to diabetes-associated hypertension are poorly understood and preclude the use of new therapeutics. 

CKD-associated glomerulo-tubular imbalance results in reduced proximal sodium (Na+) reabsorption and increased Na+ delivery and reabsorption in the distal tubule [7], and contributes to the development of hypertension [8]. The sodium-potassium-chloride cotransporter (NKCC2) and the sodium-chloride cotransporter (NCC) are expressed in the thick ascending limb of the loop of Henle and the late distal convoluted tubule, respectively. In contrast, the epithelial sodium channel (ENaC) is expressed in the distal tubule, connecting the tubule and the collecting duct, allowing Na+ entry across the luminal membrane [9,10,11,12], while the sodium-potassium-ATPase (Na-K-ATPase) expressed in the basolateral membrane within each segment of the nephron provides the driving force for the transcellular movement of sodium [13]. ENaC regulates blood pressure by fine-tuning distal tubular Na+ reabsorption [14,15]. Renal ENaC is activated by various proteases, including cathepsin B [16,17]. Cathepsin B is a cysteine protease that participates in numerous physiological and pathophysiological processes. The activity of cathepsin B is elevated in the urine of type 2 diabetic patients with varying stages of albuminuria [18]. However, the role of cathepsin B in the pathophysiology of hypertension in the diabetic kidney is incompletely understood. 

Metformin is an antidiabetic medication for patients with type 2 diabetes mellitus; it was granted FDA approval in 1994 [19]. However, the mechanisms underlying its benefits are incompletely understood [20]. Current data suggest that metformin has only a minor insignificant effect on blood pressure in nondiabetic hypertensive individuals [21]. Metformin activates the AMP-activated kinase and reduces high sodium-induced upregulation of ENaC in human umbilical vein endothelial cells [22]. However, it is unclear whether metformin regulates ENaC in renal cells and alleviates blood pressure. 

Since the development of high blood pressure can exacerbate diabetic nephropathy, we evaluated the ability of metformin to reduce blood pressure in hypertensive diabetic db/db mice. We observed that metformin normalized systolic blood pressure. Metformin treatment attenuated cathepsin B expression, which was associated with a reduction in ENaC expression and lowering of microalbuminuria. Additionally, we observed that urinary extracellular vesicles (EVs) are carriers of active cathepsin B, which was mitigated by metformin treatment. Collectively, our data provide new insight into the pathogenesis of diabetic nephropathy accompanied by hypertension and a novel understanding of ENaC regulation by metformin to mitigate hypertension-induced diabetic kidney injury.

## 2. Materials and Methods

### 2.1. Animal Studies

Male and female db/db mice (BKS.Cg-Dock7m +/+ Leprdb/J; Stock No: 000642) and cathepsin B knockout mice (B6; 129-Ctsb; Stock No. 030971) were purchased from the Jackson Laboratory (Bar Harbor, ME). All animal studies were performed under an approved University of Florida Institutional Animal Care and Use Committees protocol (#201909084) and were in compliance with the National Institutes of Health “Guide for the Care and Use of Laboratory Animals.”

### 2.2. Gel Diet

A regular normal salt diet (NSD) or 4% high salt diet (high salt diet (HSD)) was prepared using type 1 water, agar, NaCl, and powdered base diet (Tekland TD.94045.PWD; Envigo; Indianapolis, IN, USA). The gel diet was poured into food cups and stored at 4 °C for up to 3 days.

### 2.3. Metabolic Cage Studies and Administration of Metformin and Vehicle

Male and female db/db mice were kept on a NSD. At 16 weeks of age, they were switched to a HSD for 7 days to induce hypertension. Metformin (dose of 60mg/kg of body weight per day) or vehicle (0.9% normal saline solution) were administered by oral gavage (*N* = 8 each) between 9am-noon once a day for 4 days after the mice developed hypertension from being maintained on the HSD. Water consumption and urine output were measured daily before and after treatment with metformin or vehicle (saline). 

### 2.4. Tail Bleeds for Measuring Blood Glucose

The blood was collected from the tail vein in a micro-hematocrit capillary tube between 9–11am. The plasma was separated by centrifuging at 3000 rpm for 10 min and stored at −20 °C. The concentration of blood glucose was measured using a digital glucometer (CVS Health, Woonsocket, SD, USA).

### 2.5. Blood Pressure

Systolic blood pressure was measured as previously described by our group [23].

### 2.6. Albuminuria Measurements

Urine was collected over 24 h in a metabolic cage. Albuminuria was measured using the Exocell Kit (Ethos Biosciences, NJ, USA) using the manufacturer’s instructions and presented as milligram of albumin per gram of creatinine, as previously described by Scindia et al. [24].

### 2.7. Histology

Five-micron thick formalin-fixed kidney sections from vehicle or metformin treated db/db mice were paraffin embedded, and Trichrome staining was used to assess histopathology using the University of Florida core facility. Images were acquired on a Nikon eclipse Ts2R microscope (Melville, NY, USA). 

### 2.8. Cell Culture and Transepithelial Current Measurements

Mouse cortical collecting ducts (mpkCCDc14) were plated on Transwell permeable supports (Corning) and maintained in DMEM-F12 media supplemented with 50 nM dexamethasone, 1 nM triiodothyronine, 2 mM L-glutamine, 0.1% penicillin-streptomycin, 20 mM HEPES, and 10% exosome depleted FBS at 37 °C in 5% CO_2_. Transepithelial voltages and resistances were measured in a confluent monolayer of the vehicle or metformin-treated cells using an epithelial voltmeter (EVOM2; World Precision Instruments, Sarasota, FL, USA). At the end of the experiment, 0.5 μM amiloride was added to the top side of the Transwell supports, and the transepithelial current was calculated from Ohm’s law.

### 2.9. Immunohistochemistry 

The paraffin-embedded tissue sections from vehicle or metformin treated db/db mice were used for staining. After deparaffinization (Xylene (twice, 5 min), 100% ethanol (twice, 5 min), 95% ethanol (twice, 5 min), 70% ethanol (once, 5 min), 50% ethanol (once, 5 min), and type one water, the tissue was boiled in 10 mM sodium citrate buffer (Vector labs) for 20 min. After a PBS wash, blocking was performed with 2.5% normal horse serum (Vector labs) at room temperature. Next, the tissue sections were incubated with cathepsin B antibody ((1:500) Cell Signaling Tech, 3383) for 2 h and incubated with a fluoresceine-labeled horse anti-rabbit secondary for 1 hr. After washing with PBS, slides were mounted using Vectashield anti-fade mounting media. Fluorescence images were captured using an Eclipse Ti2 by Nikon Instruments Inc., Melville, NY, USA.

### 2.10. Western Blotting

Snap-frozen kidneys were used for protein analysis. Tissue lysates were prepared in tissue protein extraction reagent (Thermo Fisher Scientific; Waltham, MA, USA) containing Halt protease and phosphatase inhibitors (Thermo Fisher Scientific) using an Omni TH homogenizer (Warrenton, VA, USA). The tissue lysates were centrifuged at 13,000 rpm at 4 °C for 30 min, and the supernatant was sonicated twice for 10 s intervals while on ice. Protein concentration was determined using a Bicinchoninic acid protein assay (Thermo Fisher Scientific). Fifty micrograms of protein were loaded onto 4–20% Tris·HCl polyacrylamide gels and resolved using the Criterion electrophoresis system (BioRad; Hercules, CA, USA). The resolved proteins were electrically transferred onto nitrocellulose blotting membranes (GE Healthcare, Piscataway, NJ, USA) using the Criterion transfer system (Bio-Rad). After blocking with 5% nonfat milk 1× Tris-buffered saline (1×TBS) (Bio-Rad), the membranes were incubated with primary antibodies (anti-ENaC alpha 59 antibody [25], cathepsin B antibody (Cell Signaling Tech, 3383), anti-HSP70 antibody (4872; Cell Signaling; Danvers, MA, USA), caveolin-1 antibody (3267; Cell Signaling), annexin A2 antibody (8235; Cell Signaling), and lamin A/C antibody (4777; Cell Signaling) at a dilution of 1:1,000 in BSA in 1× TBS (5% wt/vol) or actin HRP antibody (A3854; Sigma-Aldrich, St. Louis, MO, USA) at a dilution of 1:20,000 while on a rocker at 4°C overnight. The membranes were then incubated with horseradish peroxidase-conjugated goat anti-rabbit secondary antibody at a dilution of 1:3000 prepared in blocking solution while on a rocker at room temperature for 1 h. The membranes were developed with SuperSignal West Pico reagent (Thermo Scientific) for 5 min, and then imaged on a Bio-Rad imager. 

### 2.11. Isolation of Urinary Extracellular Vesicles

Urine was centrifuged at 1000× *g* for 10 min. The supernatants were filtered with a 0.22 μm Nalgene filter (Thermo Fisher Scientific), and centrifuged at 10,000 g for 30 min. This supernatant was then subject to ultracentrifugation at 118,000 g for 70 min at 4°C using a fixed-angle Ti-70 rotor (Beckman Coulter, Brea, CA, USA). The extracellular vesicle pellets were resuspended in ultrapure 1× PBS (0.22 μm filtered) and subjected to a second round of ultracentrifugation at 118,000 g for 70 min at 4°C. The pelleted EVs were reconstituted in ultrapure 1× PBS. 

### 2.12. Extracellular Vesicle Characterization

Extracellular vesicles were characterized by Western blotting for multiple EV protein markers, including anti-HSP70, caveolin-1, annexin A2, and the non-EV marker lamin A/C. Transmission electron microscopy was used to visualize the EV preparations as previously described by our group [26]. 

### 2.13. Nanoparticle Tracking Analysis

The concentration of the urinary EVs was determined by Nanoparticle Tracking Analysis (NTA) using an NS300 machine equipped with NTA 3.4 Build 16 software. 

### 2.14. Electrolyte and Osmolality Analysis

Urinary electrolytes (sodium, potassium, and chloride) were measured using an electrolyte analyzer (SmartLyte, Diamond Diagnostics; Holliston, MA, USA) after the urine samples were diluted with urine diluent (Diamond Diagnostics). Urine osmolality was measured using an auto-sampling turntable model 2430 osmometer (Precisions Systems Inc; Natick, MA, USA). 

### 2.15. Labeling of EVs for Cellular Uptake Assays

The Claret Far Red fluorescent Cell linker kit (Sigma; St. Louis, MO, USA) was used to label freshly isolated urinary EVs. Cholera Toxin Subunit B conjugated to Alexa Fluor 488 (Thermo Fisher Scientific; Waltham, MA, USA) was used to label lipid rafts of mpkCCD cells. The uptake of the EVs was visualized using a widefield Nikon Ti-E inverted microscope. 

### 2.16. Statistical Analysis

A Student *t*-test was used to determine statistical significance between two groups, while a one-way ANOVA was used to determine statistical significance for more than two groups. GraphPad Prism 9 (Boston, MA, USA) was used for all statistical analyses. The values presented are expressed as means ± SEM.

## 3. Results

### 3.1. Metformin Decreases Systolic Blood Pressure and Increases Urinary Sodium Levels in Hypertensive Diabetic Mice 

**Figure 1:**
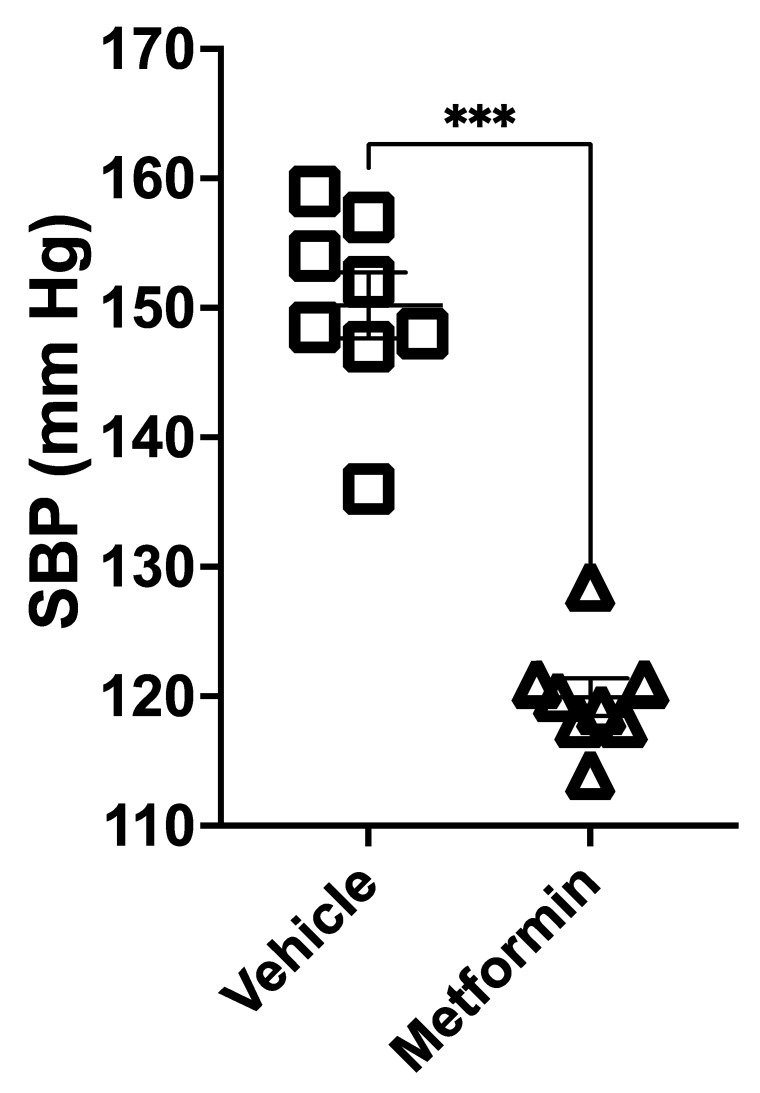
Metformin alleviates systolic blood pressure measurements in diabetic db/db mice on a high salt diet (HSD). The tail-cuff method was used to measure blood pressure in male and female salt-loaded diabetic db/db mice maintained on an HSD and treated with vehicle or metformin. *N* = 8. Data are plotted as mean ± SEM, and *** represents a *p*-value < 0.001.

**Figure 2:**
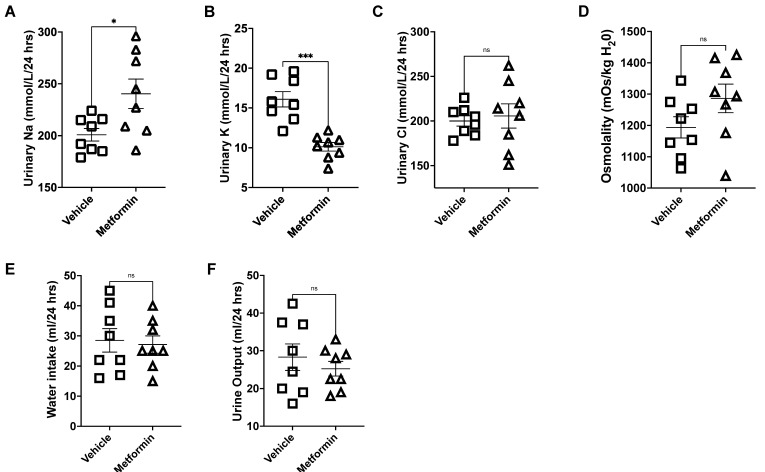
Analysis of urinary sodium, potassium, chloride, osmolality, water intake, and urine output from vehicle- or metformin-treated db/db mice maintained on an HSD. (**A**) Urinary sodium (mmol/L/24 h); (**B**) urinary potassium (mmol/L/24 h); (**C**) urinary chloride (mmol/L/24 h); (**D**) urinary osmolality (mOsm/Kg H20); (**E**) water intake (ml/24 h); (**F**) urine output (ml/24 h). *N* = 8. Data are plotted as mean ± SEM, * represents a *p*-value < 0.05, and *** represents a *p*-value < 0.001. ns: not significant.

Compared to vehicle, metformin treatment decreased blood glucose in hypertensive diabetic db/db mice on a high salt diet (HSD; 4% NaCl) (Table 1). The development of hypertension after salt loading db/db mice has been previously reported [27]. Metformin treatment ameliorated the HSD-induced increase in the systolic blood pressure in db/db mice (Figure 1). Increased urinary sodium retention is a hallmark of hypertensive kidney disease. Urinary sodium excretion in diabetic db/db mice kept on an HSD was significantly increased by metformin treatment (Figure 2A), while urinary potassium excretion was significantly reduced (Figure 2B). Urinary chloride (Figure 2C), urine osmolarity (Figure 2D), water intake (Figure 2E), and urine output (Figure 2F) were comparable. 

### 3.2. Metformin Reduces HSD-Induced Glomerular Injury in db/db Mice

**Figure 3:**
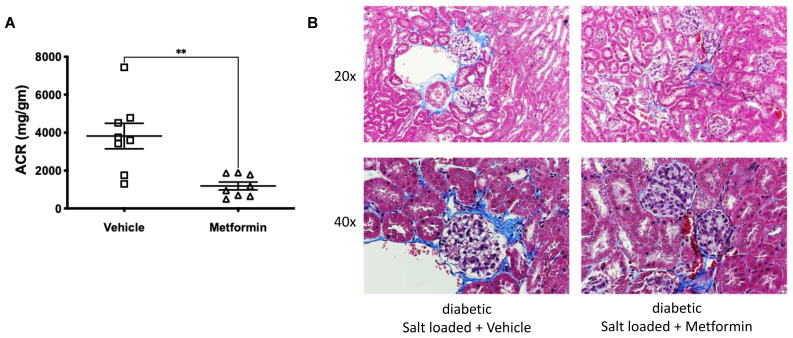
Metformin delays onset on glomerular injury in db/db mice on an HSD. (**A**) Glomerular injury as indicated by microalbuminuria (ACR) in diabetic db/db mice maintained on an HSD and treated with vehicle or metformin: ** *p* < 0.01. (**B**) Renal histology (H & E) and trichrome staining show glomerular hypertrophy and hypercellularity in the vehicle-treated mice. These pathological changes were reduced by metformin treatment.

The glomerular injury was assessed in the two groups of mice. Diabetic db/db mice on an HSD had significant glomerular injury as measured by urinary microalbuminuria (Figure 3A), which was significantly attenuated by metformin (Figure 3A). Renal histology (trichrome staining) corroborated the observed microalbuminuria (Figure 3B). Trichrome staining of the diabetic db/db mice on the HSD showed blue collagen deposits in the periglomerular regions, which were reduced in the metformin-treated group (Figure 3B). Reduced ACR and periglomerular collagen deposits collectively indicate that metformin treatment ameliorates glomerular injury in settings of hypertension and diabetes.

### 3.3. Metformin Reduces Protein Expression of the Protease Cathepsin B and Proteolysis of ENaC Alpha Subunit 

**Figure 4:**
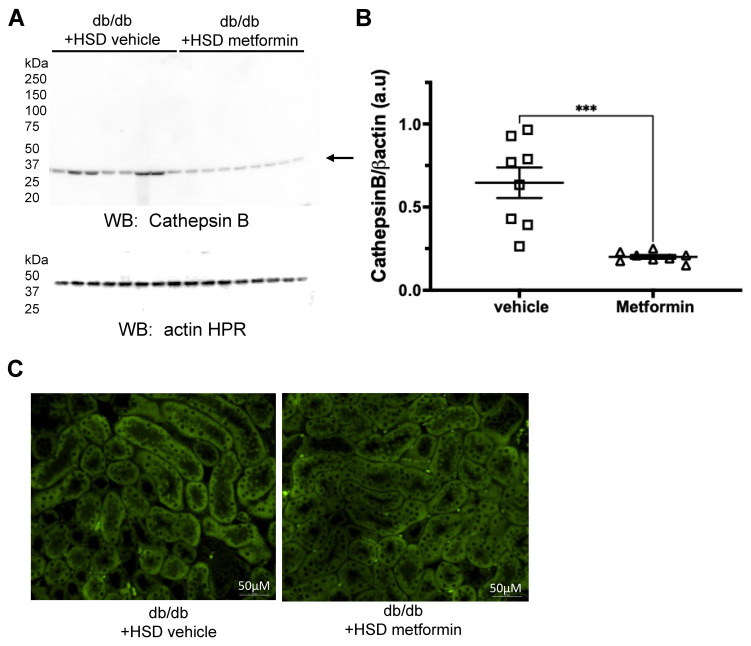
Renal expression of cathepsin B protein is attenuated by metformin treatment. (**A**) Western blot for cathepsin B protein expression in kidney cortex lysates from vehicle-treated or metformin-treated diabetic db/db mice maintained on an HSD. (**B**) Densitometric analysis of the immunoreactive band in panel A normalized to actin (**C**) Immunohistochemistry showing cathepsin B protein expression in the kidneys of hypertensive diabetic db/db mice treated with vehicle or metformin. *N* = 8. Data are plotted as mean ± SEM, *** represents a *p*-value < 0.001, and a.u. represents arbitrary units.

**Figure 5:**
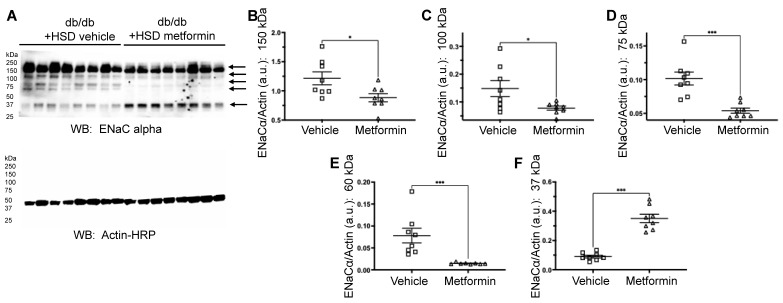
Metformin reduces the expression of renal ENaC alpha in db/db mice on an HSD. (**A**) Western blot for ENaC alpha subunit in kidney cortex lysates from vehicle- or metformin-treated diabetic db/db mice maintained on an HSD. (**B**–**F**) Densitometric analysis of the 150 kDa, 100 kDa, 75 kDa, 60 kDa, and 37 kDa immunoreactive bands indicated by arrows in (**A**). *N* = 8 for each group. Data are plotted as mean ± SEM, * represents a *p*-value < 0.05, and *** represents a *p*-value < 0.001.

Renal ENaC regulates blood pressure by fine-tuning distal tubular Na+ reabsorption. Cathepsin B is known to cleave and activate renal ENaC [16,17]. Since metformin treatment was associated with improved blood pressure and increased sodium excretion, we investigated the relevance of the cathepsin B-ENaC axis in metformin-mediated protection. Compared to vehicle-treated mice on an HSD, cathepsin B protein expression was significantly reduced in the metformin group (Figure 4A–C). The ENaC alpha subunit shows multiple bands [25], which were detected and quantified. Analysis of these unprocessed and proteolytically cleaved products of the ENaC alpha subunit revealed 37 kDa, 60 kDa, 75 kDa, 100 kDa, and 150 kDa immunoreactive bands (Figure 5A) for mice in both of the groups. Compared to vehicle-treated mice on an HSD, metformin treatment was associated with a significant reduction in the 60 kDa, 75 kDa, 100 kDa, and 150 kDa bands corresponding to the ENaC alpha subunit (Figure 5A–E).

### 3.4. Unique Profiles of Urinary EVs from Diabetic db/db Mice Treated with Metformin

**Figure 6:**
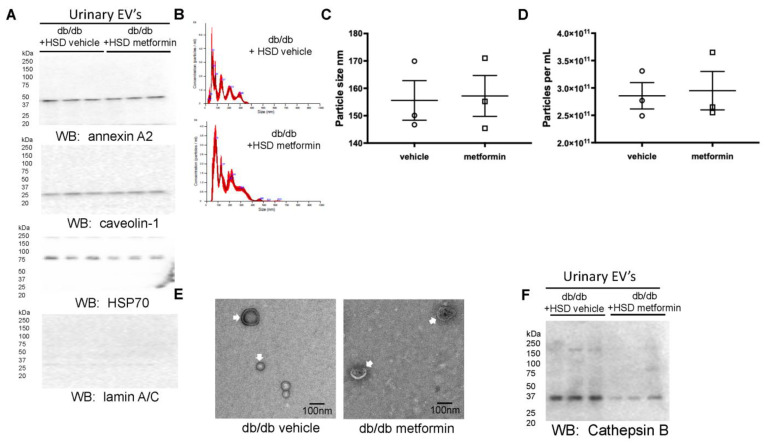
Characterization of urinary EVs from vehicle- or metformin-treated db/db mice on an HSD. (**A**) Western blot for the presence of the EV markers annexin A2, caveolin-1, HSP-70, and the absence of the non-EV protein lamin A/C. (**B**) Nanoparticle tracking analysis showing EV peak profiles from diabetic db/db mice maintained on an HSD and treated with vehicle or metformin. (**C**) Summary plot showing EV size in nanometers (nm) in each group. (**D**) Summary plot showing EV concentration in particles/mL in each group (**E**) Transmission electron microscope micrographs of pooled EVs from each group showing the absence of aggregated proteins and the presence of EVs less than 200 nm in diameter. (**F**) Western blot depicting the enrichment of cathepsin B in urinary EVs from each group. *N* = 3 EV preparations per group.

We have previously shown that EVs transfer active cargo between cells [28]. Urinary EVs were isolated from vehicle- or metformin-treated db/db mice on an HSD. The presence of the EV markers annexin A2 at 37 kDa, caveolin-1 at 25 kDa, HSP70 at 70 kDa, and the absence of a band for the non-EV marker lamin A/C at 70 kDa are shown in Figure 6A. Nanoparticle tracking analysis showed unique peak profiles of urinary EVs from the vehicle- and metformin-treated diabetic db/db mice maintained on an HSD (Figure 6B). The urine of both groups contained comparable sizes and numbers of EVs (Figure 6C,D). Transmission electron microscopy showed the absence of aggregated proteins or large EVs such as apoptotic bodies (Figure 6E). The cathepsin B levels in the EVs of metformin-treated mice on an HSD were significantly lower than vehicle-treated mice on an HSD (Figure 6F).

### 3.5. Urinary EVs from db/db Mice Treated with Metformin Compared to Vehicle Decrease Amiloride-Sensitive Transepithelial Current in mpkCCD Cells

**Figure 7:**
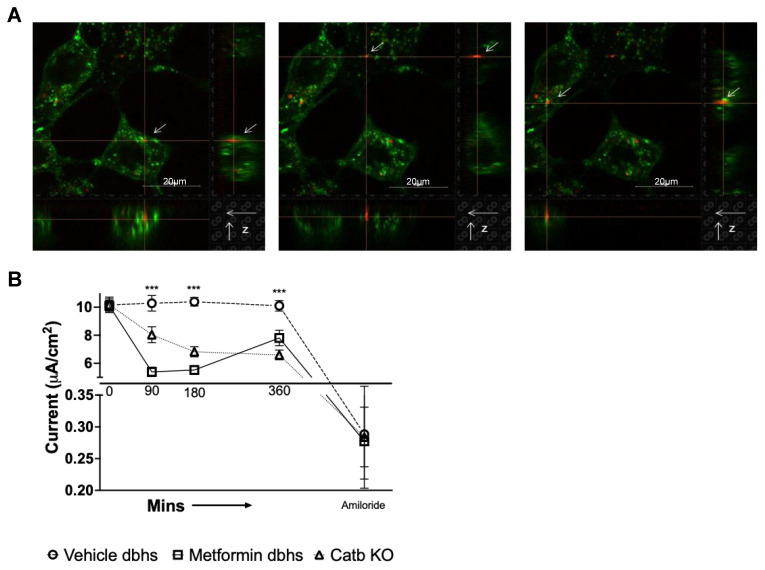
Uptake of urinary EVs from diabetic db/db mice by mouse mpkCCD cells and changes in amiloride-sensitive transepithelial current after mpkCCD cells were challenged with urinary EVs from vehicle- or metformin-treated db/db mice on an HSD. (**A**) Immunofluorescence images of mpkCCD cells (green, orthogonal view) treated for 15 min with red fluorescently labeled urinary EVs isolated from diabetic db/db mice. (**B**) Amiloride-sensitive transepithelial current measurements showing transepithelial current in mpkCCD cells over time after treating the cells with urinary EVs from either diabetic db/db mice maintained on an HSD and treated with vehicle (vehicle) or metformin (metformin), or cathepsin B knockout mice (Catb KO). *N* = 6 inserts per group, and *** represents a *p*-value < 0.001. Red fluorescence shows urinary EVs labeled with Claret far red. Green fluorescence shows mpkCCD cells incubated with 1μg/mL of cholera toxin subunit B Alexa Fluor 488 conjugate.

We next investigated the ability of EVs to induce a phenotypic change in mouse collecting duct cells. The mpkCCD cells, a mouse cortical collecting duct cell line, were used for these studies. The plasma membrane of the mpkCCD cells was labeled with a green fluorescent cholera toxin subunit B Alexa Fluor 488 conjugate. This was followed by addition of 1.5 × 10^6^ particles/mL of fluorescently labeled EVs (pooled from four diabetic db/db mice) for 15 min. As shown in Figure 7A, EVs were avidly taken up and localized to the plasma membrane of the mpkCCD cells. Next, we investigated whether urinary EVs isolated from HSD-fed diabetic mice treated with vehicle or metformin can alter amiloride-sensitive transepithelial current over time. Urinary EVs isolated from cathepsin B knockout (KO) mice were used as controls. We challenged the luminal side of mpkCCD cells with 2.7 × 10^8^ EV particles/mL isolated from the three groups of mice and measured changes in amiloride-sensitive transepithelial current over time. Urinary EVs from metformin-treated db/db mice or cathepsin B knockout mice significantly reduced transepithelial current in mpkCCD cells over time, compared to urinary EVs from vehicle-treated db/db mice (Figure 7B). 

## 4. Discussion

We identified the ability of metformin to alleviate hypertension in settings of diabetes. The HSD in diabetic mice was associated with abundant protein expression of the amiloride-sensitive epithelial sodium channel and cathepsin B, a protease that cleaves and activates renal ENaC. Metformin treatment significantly reduced these HSD-induced pathological features in diabetic mice. Based on the ability of metformin to reduce the density of the active form of renal ENaC, it was not surprising that metformin treatment increased urinary sodium and decreased urinary potassium. The antidiabetic drug rosiglitazone also increases urinary sodium secretion in db/db mice [29]. Next, we demonstrated that metformin treatment changes cathepsin B levels in urinary EVs without affecting their numbers and is associated with reduced ENaC activity. Urinary EVs are generated mostly by cells of the nephron. Our observations suggest that EVs act as a medium of intra-renal cellular crosstalk and provide clues about how pathology may cascade across the kidney in hypertensive diabetes.

The effect of metformin in C57BLK/6 wild-type mice has been previously investigated, and it was found to decrease renal NCC phosphorylation [30]. In contrast, Pavlov et al. [31] showed that in Dahl salt-sensitive rats neither renal ENaC activity nor mean arterial pressure was affected by chronic infusion of metformin. Our observations show an association between metformin treatment and reduced activation of ENaC and support the blood pressure lowering ability of metformin in diabetic mice on the same background (db/db mice are on C57BLK/6 background). These conflicting observations across species warrant more studies. 

Although there are several proteases that cleave and activate renal ENaC, we focused on cathepsin-B-mediated proteolysis of ENaC, as diabetic patients with T2DM show an increase in urinary excretion of cathepsin B compared to healthy individuals [18]. Cathepsin B cleaves and activates renal ENaC [16] and contributes to the development of hypertension [17]. Importantly, cathepsin B not only cleaves and activates renal ENaC but it also cleaves the myristoylated alanine-rich C kinase substrate (MARCKS) [32]. MARCKS positively regulates ENaC protein expression and activity at the luminal membrane of the distal tubule and collecting duct cells [25,33,34]. Furthermore, the ENaC alpha subunit can generate current by itself and is essential for overall channel activity [35]. While metformin is a drug of choice for treatment of T2DM, our findings identify its novel blood pressure reducing effects, possibly in part by altering cathepsin B protein expression and thus ENaC levels. 

Our data on urinary EVs further support this hypothesis. We show for the first time that metformin treatment reduces the cathepsin B content of urinary EVs derived from hypertensive diabetic mice. While cathepsin B is a lysosomal protein that is present in most cell types [36], urinary EVs act as a medium for intercellular cargo transport (including cathepsin B) [28,37]. In healthy distal tubule epithelial cells, ENaC activity appears to be negatively regulated by EVs isolated from healthy proximal tubule cells [28]. Our data show that urinary EVs are rapidly taken up by normal mouse cortical collecting duct cells. Importantly, the transepithelial current in mpkCCD cells was significantly lower after treating the cells with urinary EVs isolated from metformin-treated db/db mice or from cathepsin B knockout mice compared to cells treated with urinary EVs from vehicle-treated hypertensive diabetic db/db mice. These in vitro results support our in vivo observations. 

A limitation of our current study is that we did not investigate the regulation of other proteases that are known to cleave and activate ENaC. We focused on cathepsin B since our previous studies showed extracellular vesicles contain this protease [38]. Another limitation of our study is that we did not examine other mechanisms in the proximal segment of the nephron that may play a role in the pathophysiology of hypertension in the diabetic kidney. 

**Figure 8:**
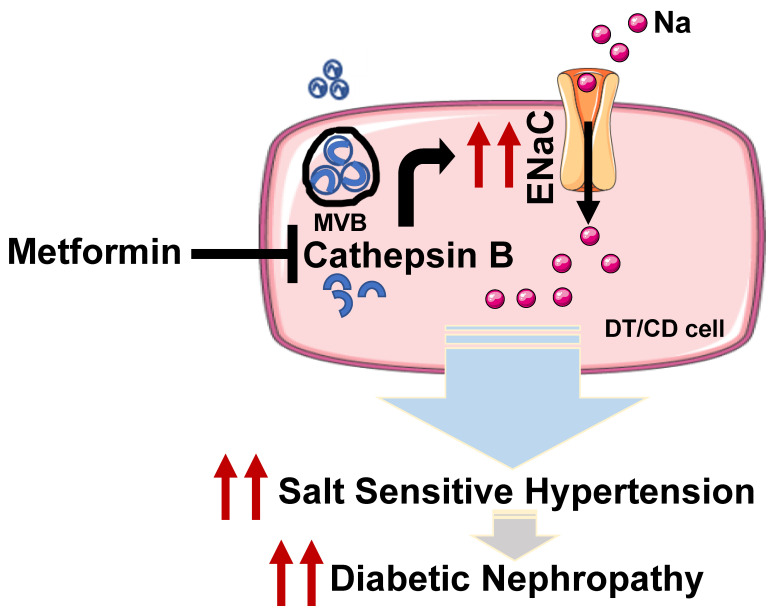
Mechanistic model showing a putative role for metformin in inhibiting renal NCC and ENaC activation and reducing blood pressure in the diabetic kidney. An increase in cathepsin B mediated proteolysis of ENaC alpha in distal tubule (DT) and collecting duct (CD) cells, contributes to the development of salt-sensitive hypertension in the diabetic kidney. Hypertension exacerbates the development of diabetic nephropathy. Metformin attenuates Cathepsin B levels in the kidneys and in EVs to alleviate salt-sensitive hypertension in the diabetic kidney. In addition, metformin reduces the density of ENaC at the luminal membrane. MVB refers to multivesicular body.

Taken together, these data show a beneficial role for metformin in reducing high blood pressure in settings of diabetes. We identified a novel mechanism by which metformin may alleviate high blood pressure by attenuating cathepsin B levels in the kidney and in EVs to reduce ENaC activity (Figure 8, graphical representation). Future studies are needed to identify microRNAs, proteins, and metabolites within these EVs that may also contribute to the negative regulation of renal ENaC. 

## Figures and Tables

**Table 1:** Blood glucose levels in vehicle- or metformin-treated db/db mice maintained on a high salt diet (HSD) (*N* = 8).

Groups	Mean	SEM
HSD+ Vehicle	>33.33 mmol/L	NA
HSD+ Metformin	24.74 mmol/L	1.90 mmol/L

## Data Availability

The individual data points of each dataset are shown within the plots.
